# The validity of the Royal College of Pathologists’ colorectal cancer minimum dataset within a population

**DOI:** 10.1038/sj.bjc.6604036

**Published:** 2007-10-16

**Authors:** N J Maughan, E Morris, D Forman, P Quirke

**Affiliations:** 1 Pathology & Tumour Biology, Leeds Institute for Molecular Medicine, St James's University Hospital, University of Leeds, Level 4 Wellcome Brenner Building, Beckett Street Leeds, Leeds LS9 7TF, UK; 2 Cancer Epidemiology Group, Centre for Epidemiology and Biostatistics, The University of Leeds, Arthington House, Cookridge Hospital, Leeds LS16 6QB, UK; 3 Northern & Yorkshire Cancer Registry and Information Service, Arthington House, Cookridge Hospital, Leeds LS16 6QB, UK

**Keywords:** colorectal neoplasms, minimum dataset, histopathology, staging

## Abstract

Quality of colorectal cancer pathology reports is related to individual patient prognosis and future treatment options. This study sought to validate the prognostic utility of the Royal College of Pathologists minimum pathology dataset (MPD), regarded as the ‘gold standard’, within a population. Retrospective study of the survival of 5947 surgically resected colorectal cancer patients for whom an MPD had been collected. Variables were related to survival. The study population was representative of the Yorkshire colorectal cancer population. Survival was poorer in older patients and colonic tumours and improved over the study period. Local invasion, total number of lymph nodes retrieved, nodal stage, extramural vascular invasion, peritoneal involvement, distance of invasion beyond the muscularis propria, and in rectal cancers, circumferential resection margin involvement and distance to this margin were all validated as of prognostic significance within a population. Failure to report extramural vascular invasion, peritoneal involvement or circumferential resection margin status was associated with a worse survival than absence of the factor. All variables within the Royal College of Pathologists MPD are of prognostic significance. High-quality pathology reports are essential in providing accurate prognostic information and guiding optimal patient management.

High quality histopathological reporting is vital in the management of colorectal cancer. Assessment of the surgical specimen determines the stage of disease, the completeness of the surgical excision and, hence, the prognosis and future treatment options for the patient. It is crucial, therefore, that pathology reports contain all the accurate information required to fulfil these functions.

Many pathological features have been identified as being of prognostic value and therapeutic significance and, as a consequence, there are numerous guidelines stating which pathological features should be reported for colorectal cancer (Henson *et al*, 1994). In 1998 the Royal College of Pathologists synthesised, through the available literature and expert consensus, the features they deemed as being most important for determining the prognosis of colorectal cancer into a minimum pathology dataset (MPD) (Quirke and Williams, 1998). The document is a proforma detailing the minimum data items a pathologist should record when reporting colorectal cancer tumours. Much of the evidence through which it was formulated, however, originated from small single-centre studies or specialised trial environments and its validity has never been tested in a population-based setting.

The Northern and Yorkshire Cancer Registry (NYCRIS), in collaboration with Yorkshire's pathologists, first published a proforma for the pathological reporting of colorectal cancer resections in 1995 following a decision to standardise the collection of pathology data for registry use. The NYCRIS proforma was largely adopted by the Royal College of Pathologists as their MPD in 1998 and so the Northern and Yorkshire region is in the unique position of having access to identical data to that on this MPD from 1995. The NYCRIS proforma includes all MPD items and a few additional data points. NYCRIS also collects basic demographic and survival information about all patients diagnosed with cancer in the region. It is, therefore, possible to link the MPD to the survival data and, hence, assess the prognostic ability of the minimum dataset items. This study sought to determine the prognostic value of the contents of the Royal College of Pathologists MPD in a population of 3.6 million.

## MATERIALS AND METHODS

All colorectal cancer patients for whom a MPD was completed, therefore having received a surgical resection of their tumour, and submitted to NYCRIS between 1995 and 2000 were identified. Routinely recorded information about these patients’ disease and its management were then downloaded from the main registry database and the two datasets merged. Any discrepancies were resolved by review of the original pathology report. Patients with multiple colorectal cancers were excluded. Cases without an MPD were also identified and their overall survival compared to exclude bias due to a failure to return forms.

The survival time for each patient was calculated from date of surgery to date of death from all causes or when censored (9 February 2006). Kaplan–Meier curves were created to compare univariate survival and log–rank tests were used to test for statistical significance. Cox-proportional hazards models were used to determine the impact of sex and age on the survival estimates for each prognostic factor.

Variables assessed were extent of local invasion, number of nodes retrieved, nodal stage, extramural vascular invasion (EMVI) and peritoneal involvement for all patients and circumferential margin involvement (CRM) and distance to the CRM for rectal tumours only. Distance of invasion beyond the muscularis propria was an additional item included on the Yorkshire form but omitted from the Royal College MPD and the prognostic significance of this variable was also investigated to support its inclusion in any future revision. Tumour grade was not investigated as poor correlation was found between colorectal cancer and main registry databases due to changes in the coding system over time, meaning accurate allocation to present groupings of well, moderate and poor differentiation was impossible.

## RESULTS

Between 1995 and 2000, 5947 colorectal pathology forms were submitted to NYCRIS. This represents 55.5% of the entire surgically managed colorectal cancer population in Yorkshire over that time period. There was no significant difference between the observed five-year survival of our study population (48.5%: 95% CI 47.2–49.8%) and the entire Yorkshire cohort of surgically treated colorectal cancer patients identified within the main cancer registry database (46.7%: 95% CI 45.8–47.6%).

The characteristics and five-year survival of our study population is presented in Table 1. There was a significant difference in survival observed across cancer sites, with colon cancers having a survival of 45.9% (95% CI 44.2–47.5%) and rectal cancers 53.4% (95% CI 51.0–55.7%). Survival was poorer in the older age groups and improved over the study period from 44.1% (95% CI 39.6–48.4%) in 1995 to 51.8% (95% CI 48.9–54.7%) in 2000.

### Local invasion

**Figure 1:**
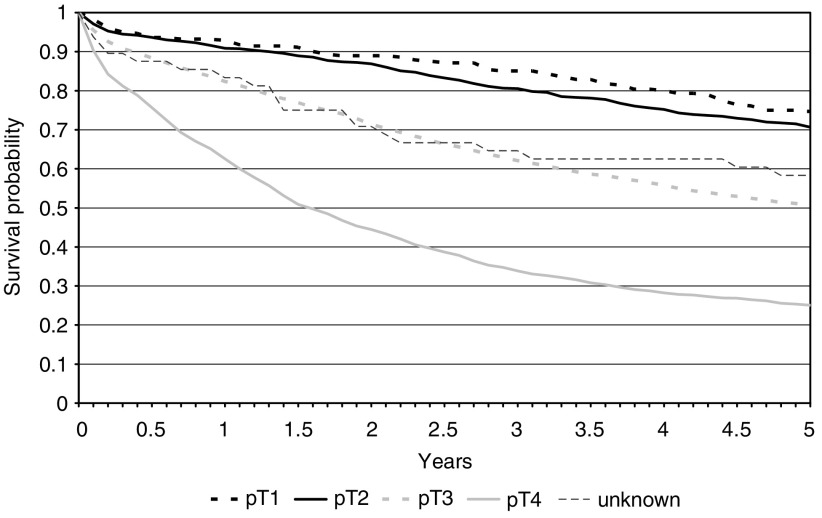
Kaplan–Meier survival curves of the colorectal cancer population by pathological T stage.

Figure 1 shows the five-year survival curves of patients in each of the pathological T stages. Patients with T1 (74.6%: 95% CI 69.1–79.3%) and T2 tumours (70.7%: 95% CI 67.5–73.6%) had significantly better survival than those with T3 (50.6%: 95% CI 48.9–52.2%) or T4 tumours (25.1%: 95% CI 22.9–27.3%). Patients in whom the extent of local invasion was not recorded had an intermediate five-year survival of 58.3% (95% CI 43.2–70.8%). The effect remained after adjustment for patient age and gender (Table 2).

### Total number of lymph nodes retrieved

Patients from whom greater than 12 nodes were retrieved had significantly higher survival (53.0%: 95% CI 50.7–55.2%) compared to those with the lowest nodal yield (45.4%: 95% CI 43.1–47.7%) (*P*<0.01). Patients in whom the number of nodes retrieved failed to be reported, had the worst survival (38.2%: 95% CI 27.8–48.7%). Age and gender did not influence the results (Table 2)

### N stage

**Figure 2:**
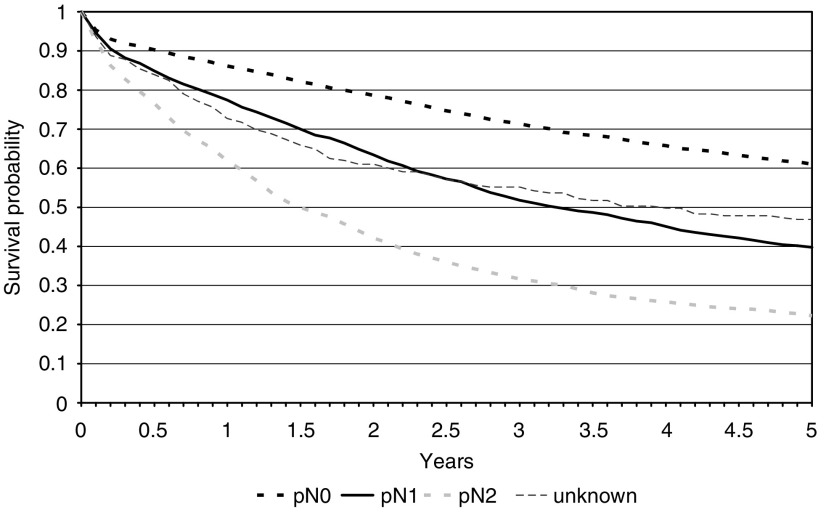
Kaplan–Meier survival curves of the colorectal cancer population by pathological N stage.

The greater the number of positive nodes identified the worse the survival of the patients (Figure 2). Those falling into the N2 category (i.e., four or more positive nodes identified) had the lowest five-year survival (22.2%: 95% CI 19.7–24.9%) compared to those with one to three (39.8%: 95% CI 37.4–42.2%) or those who were node negative (61.0%: 95% CI 59.3–62.7%). Those patients in whom the number of positive nodes was not reported had an intermediate five-year survival of 46.8% (95% CI 39.9–53.5%). The effects remained statistically significant after adjusting for age and gender (Table 2).

### Extramural vascular invasion

**Figure 3:**
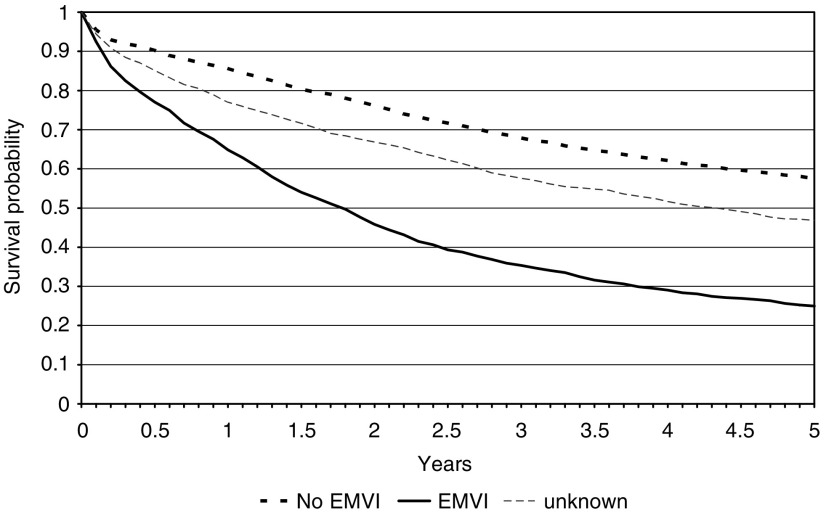
Kaplan–Meier survival curves for the colorectal cancer population by presence or absence of extramural vascular invasion (EMVI).

Figure 3 shows the presence of EMVI was also prognostic. Patients in who vascular invasion was present had a poorer five-year survival (25.0%: 95% CI 22.4–27.6%) than those in whom it was absent (57.4%: 95% CI 55.7–59.2%). Again, those in whom the feature was not reported had an intermediate survival between the two reported groups of 46.8% (95% CI 44.4–49.1%) and adjustment for age and gender did not influence the results (Table 2).

### Peritoneal involvement

**Figure 4:**
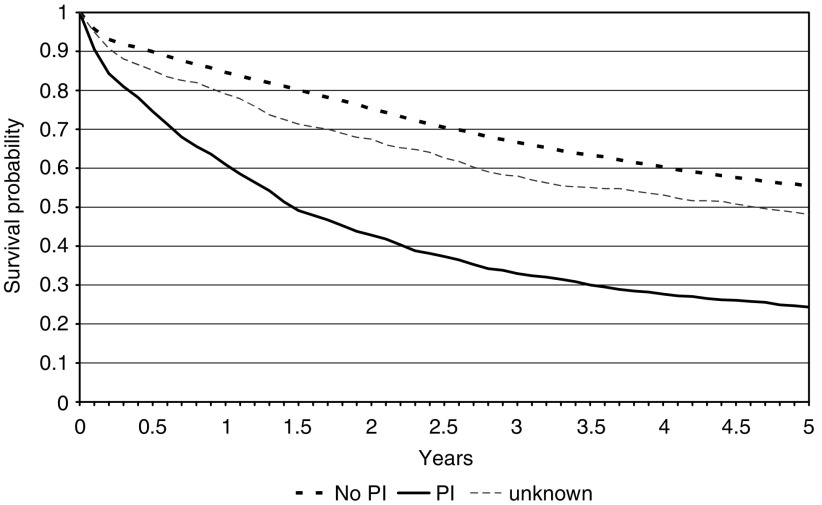
Kaplan–Meier survival curves for the colorectal cancer population by presence or absence of peritoneal involvement (PI).

A very similar effect, presented in Figure 4, was observed in relation to peritoneal involvement. Those possessing peritoneal involvement had a five-year survival of 24.3% (95% CI 21.9–26.8%) compared to 55.4% (95% CI 53.9–56.9%) in those in whom it was absent. Patients for whom the factor was not reported again had an intermediate survival between the two other groups (48.1%: 95% CI 44.3–51.8%). Again, there was no influence of patient age or gender on the results.

### Tumour perforation

In pT4 tumours those with perforation through the tumour had a worse survival (26.4%: 95% CI 19.9–33.2%) than those with no perforation (32.3%: 95% CI 26.0–38.9%) although this did not reach statistical significance due to low reporting rates of just 25.8% of this factor in pT4 tumours. Adjustment for age and gender had no impact on the results (Table 2).

### Distance of invasion beyond muscularis propria

**Figure 5:**
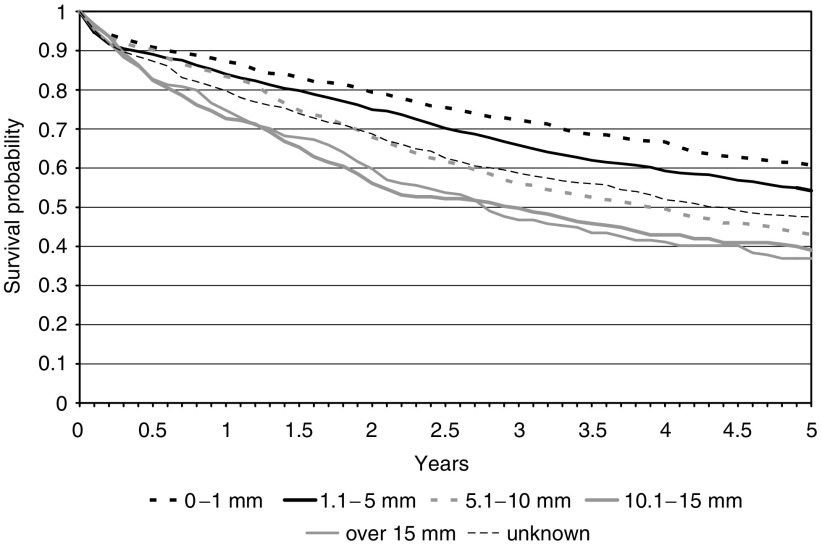
Kaplan–Meier curves for the colorectal cancer population by distance of invasion beyond the muscularis propria.

Increasing distance of invasion beyond the muscularis propria in pT3 tumours was associated with decreasing survival (Figure 5) independently of the age and gender structure of the population (Table 2). Patients who had a distance of invasion of less than 1 mm had a five-year survival of 60.8% (95% CI 57.0–64.3%) compared to 36.9% (95% CI 30.5–43.4%) for those who had a depth of invasion greater than 15 mm. Patients in whom this feature was not reported had intermediate survival at 47.5% (95% CI 43.8–51.2%).

### Circumferential resection margin involvement

**Figure 6:**
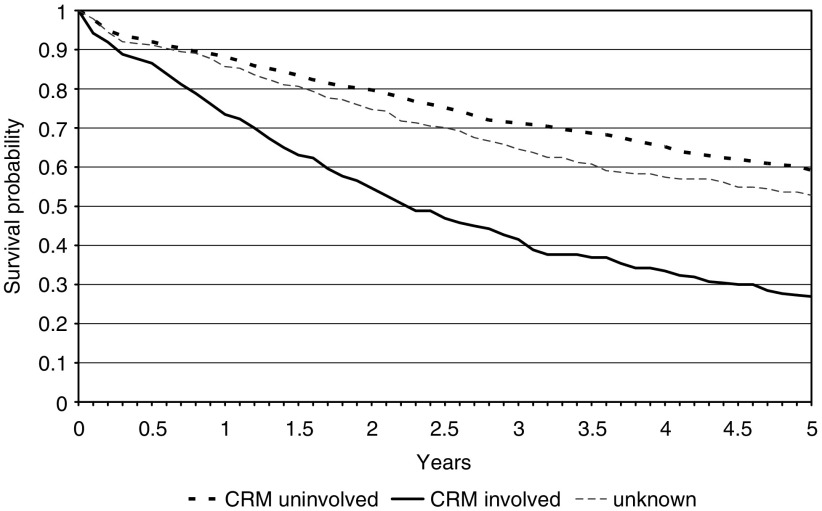
Kaplan–Meier curves for the rectal cancer population by involvement of circumferential resection margin.

The survival of rectal cancer patients with an involved CRM, as defined by the tumour lying less than one mm from the CRM, was significantly worse (26.9%: 95% CI 21.7–32.4%) than patients in whom this margin was clear (59.2%: 95% CI 56.4–61.9%)(*P*<0.01). Patients for whom this feature was not reported again had an intermediate survival of 52.7% (95% CI 46.2–58.9%). These data are presented in Figure 6. Age and gender had no effect on the trends seen (Table 2).

### Distance to circumferential resection margin

Patients in whom the distance of the tumour from the circumferential resection margin was less than 1 mm had significantly poorer survival (33.3%: 95% CI 27.1–39.6%) than those in whom the distance was greater than a millimetre (*P*<0.01). Survival at 2 mm was 52.4% (95% CI 44.0–60.0%). There was no strong trend from improved survival when patients were grouped according to increasing millimetre increments of distance to the circumferential resection margin (Table 1).

### Quality of reporting

As the total number of lymph nodes retrieved had been found to relate to survival the relationship of this factor to reporting rates of other factors was investigated (Table 3). Increasing total numbers of lymph nodes retrieved from 0 to 6 to over 12 was found to be positively correlated with detection of peritoneal involvement (*P*<0.01) and EMVI (*P*<0.01) in all cases and CRM involvement in rectal cases (*P*<0.01).

## DISCUSSION

This study provides the first evidence from a population-based setting to demonstrate that all the variables within the Royal College of Pathologists colorectal cancer minimum dataset have prognostic significance. Other variables currently not included within the minimum dataset, such as increasing distance of invasion beyond the muscularis propria in pT3 and pT4 tumours were also found to be related to decreasing survival.

### Local invasion, lymph node status, peritoneal involvement and vascular invasion

The prognostic significance of local invasion, lymph node status and lymph node yields (Pocard *et al*, 1998; Wong *et al*, 1999; Tepper *et al*, 2001; Cserni *et al*, 2002; Goldstein, 2002; Johnson *et al*, 2002; Prandi *et al*, 2002; Joseph *et al*, 2003; Pheby *et al*, 2004; Baxter *et al*, 2005; Jestin *et al*, 2005; Sarli *et al*, 2005), peritoneal involvement, tumour perforation and vascular invasion (Talbot *et al*, 1980; Shepherd *et al*, 1989; Shepherd *et al*, 1995; Shepherd *et al*, 1997; Petersen *et al*, 2002) have all previously been documented. The results of this study confirm these findings in a large population-based setting.

### Circumferential resection margins

The negative survival impact of CRM involvement has also been widely documented and our results support this previous work (Quirke *et al*, 1986; Adam *et al*, 1994; Birbeck *et al*, 2002; Wibe *et al*, 2002), but, the survival analyses looking at the distance to the circumferential resection margin in millimetre intervals indicated that there was a small and steady decrease in survival as this distance narrows. The most pronounced fall was at the one millimetre or less mark, with tumours lying between one and two millimetres from the CRM behaving similarly to those lying two to three millimetres away. This supports the work of Quirke *et al* (1986) and subsequent studies (Birbeck *et al*, 2002) but appears to contradict the more recent evidence and recommendations from the Dutch TME trial by Nagtegaal *et al* (2002).

### Distance of invasion beyond the muscularis propria

This study also validates the prognostic significance of distance of invasion of tumour beyond the muscularis propria, supporting its inclusion in the revised sixth edition of TNM classification (American Joint Committee on Cancer, 2002). This data item is not currently included in the Royal College's minimum dataset but the results of our work indicate it should be included in any future revisions.

### Quality of reporting

Patients in whom specific variables were not reported appear to have an intermediate survival between those who possessed the factor and those who did not. This suggests that absence of reporting does not necessarily mean absence of the factor. In addition, even where variables were definitely reported EMVI was only found in 17.8% of cases and peritoneal involvement in 19.5% of cases. Work from the CLASICC randomised controlled trial suggest that when reported by those with a specialist interest in gastro-intestinal pathology EMVI rates of 30% are seen. This is important as the presence of some of these pathological factors would influence an oncologist to offer adjuvant treatment. If an oncologist is not aware that a patient is potentially at risk then indicated treatment could be withheld with a concomitant increase in the risk of death. This emphasises the importance of comprehensive reporting of the minimum dataset. Previous work has highlighted problems of inadequate reporting (Bull *et al*, 1997) and our results demonstrate that failure to record key items is associated with poorer outcomes. The absence of a factor on a proforma does not equate with the prognosis of those in whom it was recorded as being absent, suggesting that in a proportion of patients it was indeed present. This was seen for EMVI, peritoneal and CRM involvement. Additionally poor rates of positive reporting of EMVI, peritoneal and CRM involvement are intimately linked to a lower number of nodes found within the NYCRIS data. Proforma reporting has been shown to improve the completeness of pathological reporting (Cross *et al*, 1998; Branston *et al*, 2002) but our results indicate significant amounts of key variables were still missing. We believe the use of computer proformas in which all data items had to be completed before a pathologist could finish a report and the careful auditing of pathology reporting against standards is essential. In addition, adequate time must be made available for pathologists to undertake thorough pathological examinations of all colorectal cancer specimens. This would improve the quality of pathological information, access of patients to adjuvant therapy and would be a good investment for cancer care.

## Figures and Tables

**Table 1:** Characteristics and five-year survival of the study population

**Characteristics**		***n***	**%**	**% five-year survival**	**95% Confidence interval**
Cancer site	Colon	3537	59.5	45.9	44.2–47.5
	Rectosigmoid	710	11.9	50.0	46.3–53.6
	Rectal	1700	28.6	53.4	51.0–55.7
Age group	<51	357	6.0	64.4	59.2–69.2
	51–60	834	14.0	60.1	56.7–63.3
	61–70	1665	28.0	55.1	52.7–57.4
	71–80	2062	34.7	46.2	44.0–48.3
	>80	1029	17.3	27.7	25.0–30.5
Year of diagnosis	1995	488	8.21	44.1	39.6–48.4
	1996	997	16.8	46.9	43.8–50.0
	1997	1099	18.5	47.3	44.3–50.2
	1998	1069	18.0	47.2	44.2–50.2
	1999	1151	19.4	50.8	47.9–53.7
	2000	1143	19.2	51.8	48.9–54.7
pT stage	pT1	280	4.7	74.6	69.1–79.3
	pT2	832	14.0	70.7	67.5–73.6
	pT3	3372	56.7	50.6	48.9–52.2
	pT4	1415	23.8	25.1	22.9–27.3
	Unknown	48	0.8	58.3	43.2–70.8
Number of nodes examined	0–6 nodes	1792	30.1	45.4	43.1–47.7
	7–12 nodes	2218	37.3	47.7	45.6–49.8
	>12 nodes	1856	31.2	53.0	50.7–55.2
	Unknown	81	1.4	38.2	27.8–48.7
pN stage	0	3168	53.3	61.0	59.3–62.7
	1	1616	27.2	39.8	37.4–42.2
	2	958	16.1	22.2	19.7–24.9
	Unknown	205	3.5	46.8	39.9–53.5
Extramural vascular invasion	Yes	1061	17.8	25.0	22.4–27.6
	No	3139	52.8	57.4	55.7–59.2
	Unknown	1747	29.8	46.8	44.4–49.1
Peritoneal involvement	Yes	1160	19.5	24.3	21.9–26.8
	No	4111	69.1	55.4	53.9–56.9
	Unknown	676	11.4	48.1	44.3–51.8
Tumour perforation (pT4 tumours only)	Yes	167	11.8	26.4	19.9–33.2
	No	198	14.0	32.3	26.0–38.9
	Unknown	1050	74.2	23.5	21.0–26.1
Distance of invasion beyond the muscularis propria (pT3 tumours only)(mm)	0–1	688	20.4	60.8	57.0–64.3
	1.1–5	1086	32.2	54.2	51.2–57.2
	5.1–10	470	13.9	43.0	38.5–47.4
	10.1–15	205	6.1	39.0	32.4–45.6
	>15	214	6.4	36.9	30.5–43.4
	Unknown	709	21.0	47.5	43.8–51.2
CRM status (rectal tumours only)	Involved	260	15.3	26.9	21.7–32.4
	Clear	1203	70.8	59.2	56.4–61.9
	Not reported	237	13.9	52.7	46.2–58.9
Distance to CRM (rectal tumours only)(mm)	⩽1	216	17.2	33.3	27.1–39.6
	1.1–2	149	11.9	52.4	44.0–60.0
	2.1–3	118	9.4	56.8	47.4–65.1
	3.1–4	97	7.7	51.6	41.2–60.9
	4.1–5	97	7.7	60.8	50.4–69.7
	5.1–10	270	21.5	58.2	52.0–63.8
	>10	308	24.5	64.3	58.7–69.4

**Table 2:** Hazard ratios for each pathological feature assessed adjusted for age and gender

**Characteristics**		**Hazard ratio**	**Lower 95% CI**	**Upper 95% CI**	***P*-value**
Local invasion	T1	1.00			
	T2	1.22	0.97	1.53	0.09
	T3	2.10	1.71	2.58	<0.01
	T4	4.35	3.53	5.36	<0.01
	Unknown	1.92	1.24	2.98	<0.01
N stage	N0	1.00			
	N1	1.77	1.64	1.91	<0.01
	N2	3.22	2.95	3.51	<0.01
	Unknown	1.57	1.31	1.88	<0.01
Extramural vascular invasion	Present	1.00			
	Absent	0.41	0.37	0.44	<0.01
	Unknown	0.55	0.50	0.60	<0.01
Peritoneal involvement	Present	1.00			
	Absent	0.42	0.39	0.45	<0.01
	Unknown	0.52	0.46	0.59	<0.01
Tumour perforation	Present	1.00			
	Absent	0.44	0.36	0.53	<0.01
	Unknown	0.56	0.47	0.67	<0.01
Distance of invasion beyond the muscularis propria (T3's only)(mm)	0–1 mm	1.00			
	1.1–5	0.67	0.58	0.77	<0.01
	5.1–10	0.83	0.73	0.94	<0.01
	10.1–15	1.03	0.89	1.20	0.67
	>15	1.24	1.02	1.50	0.03
	Unknown	1.43	1.19	1.72	<0.01
CRM status	Involved	1.00			
	Uninvolved	0.43	0.37	0.51	<0.01
	Unknown	0.51	0.41	0.63	<0.01
Number of nodes examined	0–6	1.00			
	7–12	0.95	0.88	1.03	0.21
	>12	0.85	0.79	0.93	<0.01
	Unknown	1.27	0.96	1.67	0.09

**Table 3:** Rate of reporting of peritoneal and circumferential resection margin involvement and extramural vascular invasion by total number of lymph nodes retrieved in the study population

		**Number of nodes examined**							
		**0–6**		**7–12**		**>12**		**Unknown**	
**Characteristics**		***n***	**%**	***n***	**%**	***n***	**%**	***n***	**%**
Peritoneal involvement	Yes	304	26.2	417	35.9	426	36.7	13	1.1
	No	1295	31.5	1536	37.4	1245	30.3	35	0.9
	Unknown	193	28.6	265	39.2	185	27.4	33	4.9

EMVI	Yes	215	20.3	390	36.8	450	42.4	6	0.6
	No	804	25.6	1221	38.9	1099	35.0	15	0.5
	Unknown	773	44.2	607	34.7	307	17.6	60	3.4

CRM (rectal cases only)	Yes	66	25.4	91	35.0	99	38.1	4	1.5
	No	374	31.1	456	37.9	362	30.1	11	0.9
	Unknown	98	41.4	69	29.1	54	22.8	16	6.8
